# Effects of Chemical Cross-Linking on the Structure of Proteins and Protein Assemblies

**DOI:** 10.1021/acs.analchem.5c01092

**Published:** 2025-07-08

**Authors:** Tamar Tayri-Wilk, Nir Kalisman, Uri Raviv

**Affiliations:** † Institute of Chemistry, The Hebrew University of Jerusalem, Edmond J. Safra Campus, Givat Ram, 9190401 Jerusalem, Israel; ‡ Center for Nanoscience and Nanotechnology, The Hebrew University of Jerusalem, Edmond J. Safra Campus, Givat Ram, 9190401 Jerusalem, Israel; § Institute of Life Sciences, The Hebrew University of Jerusalem, Edmond J. Safra Campus, Givat Ram, 9190401 Jerusalem, Israel

## Abstract

Chemical cross-linking is frequently used in structural biology for protein stabilization and probing protein structures, often in combination with mass spectrometry (XL-MS). These applications assume that chemical cross-linking does not significantly perturb the protein structure. Here, we directly tested this assumption by monitoring the time course of small-angle X-ray scattering (SAXS) signals from cross-linked protein samples. We investigated two common cross-linking reagents, bis­(sulfosuccinimidyl)­suberate (BS^3^) and formaldehyde (FA), with several protein systems ranging from large microtubule filaments down to globular proteins. Across all the measured protein systems, the results consistently showed that BS^3^ did not significantly change the protein structures, whereas FA induced rapid and substantial changes, including aggregation. Notably, the impact of FA was dose-dependent, with milder structural effects at the lower concentration of 0.1 wt %. Accompanying XL-MS analyses were consistent with the SAXS observations. Overall, the results recommend that in-solution cross-linking based on NHS ester chemistry should be preferred in structural studies over FA wherever possible. In cases where FA cross-linking is used, lowering the FA concentration is of clear importance, and SAXS could be used to verify the integrity of the structure.

## Graphical abstract

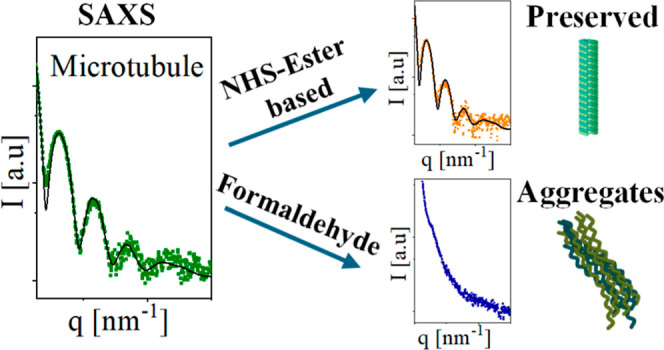

## Introduction

Structural studies of proteins employ chemical cross-linking in three main forms. First, the chemical fixation of large protein structures in cells and tissues is usually a prerequisite for their subsequent high-resolution microscopy imaging.^1,2^ Second, flexible protein regions are often stabilized by cross-linking to enable their structure determination by cryo-electron microscopy (cryo-EM).^3–5^ Finally, a combination of cross-linking and mass spectrometry (XL-MS) is frequently used to identify spatial proximities between protein regions.^6–10^

XL-MS is a powerful experimental approach for studying protein structures and interactions. In this method, proteins are incubated with a chemical cross-linking reagent that forms covalent bonds between specific amino acids. After denaturation and digestion, the cross-links are preserved as linked peptide pairs that are identified by the mass spectrometer through their unique masses. XL-MS allows high-throughput data collection, with hundreds to thousands of cross-links obtained from a single experiment. These identified cross-links are used in turn as distance restraints in integrative structural modeling in combination with other structural information sources.^11–13^ XL-MS was influential in determining the architectures of large protein complexes such as the CCT,^14^ the 26S proteasome,^15^ and the nuclear pore complex.^16^ More recently, in situ XL-MS of intact cells has provided insights into structures and interactions as they occur in the native cellular environment.^17–19^

Common to all the above applications is the assumption that chemical cross-linking does not significantly perturb the protein structure. Most XL-MS studies test this assumption indirectly by assessing the consistency of the identified cross-links with elements of the studied protein system for which atomic structures are available. Indeed, when the cross-links are mapped onto these structures, the distances between the corresponding residue pairs are within the expected span of the reagent for the majority of the cases.^20,21^ A more direct approach by Rozbeský et al.^22^ assessed the structural and functional effects of cross-linking on two globular enzymes. They have found that cross-linking reagents based on NHS ester chemistry decreased the enzymatic activity by 20–50%, but did not abolish it. They also showed by NMR that the tertiary structures of both proteins were not significantly altered. Overall, these observations supported the growing use of cross-linking in structural biology and recommended reagents based on NHS ester chemistry for their mild structural artifacts.

Given the importance of the above assumption, it is instructive to assess the structural effects of cross-linking by other approaches and examine additional protein systems and cross-linking reagents. Here, we used solution small-angle X-ray scattering (SAXS) to determine the time course of structural changes caused by cross-linking with two commonly used reagents, bis­(sulfosuccinimidyl)­suberate (BS^3^) and formaldehyde (FA). We tested both reagents on systems of varying length scales, starting from microtubule (MT) filaments, where SAXS is highly sensitive to how tubulin dimer subunits associate, and ranging to tubulin dimer subunits and other globular proteins. Although SAXS is a low-resolution method, it was nonetheless sensitive enough to measure the undesired structural effects of the different cross-linking regimes. These results further elucidate the impact of cross-linking on protein structures and recommend best practices for its use.

## Materials and Methods

### Tubulin Purification

Tubulin was purified from porcine brains using three cycles of polymerization and depolymerization, as explained.^28^ The first cycle was done at a low-salt buffer, allowing assembly in the presence of microtubule-associated proteins (MAPs). This step reduced the critical tubulin concentration for MT assembly and increased the purification yield.^54,55^ The next two cycles were performed at a high-molarity buffer to separate MAPs from the final product.^56^

### Cross-Linking Solutions

Formaldehyde (FA) and bis­[sulfosuccinimidyl] suberate (BS^3^) were purchased in powder form Sigma. A solution of 100 mM BS^3^ in Brinkley Renaturing Buffer 80 (BRB80), containing 80 mM 1,4-piperazinediethanesulfonic acid, 1 mM MgCl_2_, 1 mM EGTA, and adjusted to pH 6.9 or 7.4 with KOH, was freshly prepared before each cross-linking experiment, and added to the samples to get the desired final concentration of BS^3^. Formalin solution (37 wt % FA and 10 v/v % methanol) in water was self-prepared, and added to the samples for the desired final concentration of FA. For the XL-MS analyses, the cross-linking reactions were quenched by adding 20 mM and 500 mM TRIS-HCl (pH 8) for the BS^3^ and FA experiments, respectively.

### MT Polymerization and Cross-Linking

MTs were polymerized by incubating 10 mg/mL purified tubulin in BRB80 buffer, supplemented with 4 mM GTP, at 36 °C for 30 min. For FA cross-linking, formalin solution was diluted to either 1 or 0.1 wt % FA by the MT solution and immediately injected into the X-ray measurement quartz capillary flow-cell. For BS^3^ cross-linking, the 100 mM BS^3^ solution was diluted by the MT solution to 10 mM BS^3^ and immediately injected into the X-ray measurement quartz capillary flow-cell. All the MT solutions were measured at 36 °C.

### Supernatant Solution

The MT solution (before adding a cross-linker) was centrifuged at 20,800*g* at 36 °C for 30 min. The pellet was rich in MTs whereas the supernatant was rich in coexisting tubulin dimers and small tubulin assemblies. The top 60% of the centrifuged solution volume was cross-linked as described for the MT, and immediately injected into the X-ray measurement quartz capillary flow-cell. All the supernatant solutions were measured at 36 °C. The supernatant scattering curve at each time point was subtracted from the corresponding scattering curve of the MT solution, as explained.^31^

### Tubulin Dimer Solution

Tubulin solution at 4 °C was centrifuged at 20,800*g* at 4 °C and the top 80% was cross-linked, similarly to the MT solutions but the incubations and the X-ray measurements were performed at 4 °C. The BRB80 buffer was measured and used as background.

### Solution X-ray Scattering Measurements

Solution X-ray scattering measurements were performed in our in-house high-resolution solution small-angle X-ray scattering (SAXS) setup as described.^27^ BRB80 buffer, MT, and supernatant solutions were measured through the same spot in a flow-through capillary setup for either a 2 h exposure or 12 consecutive 10 min exposures. Between the 12 exposures, the MAR345 detector was scanned for about 2.5 min. MT and tubulin solutions with BS^3^ or FA were measured in a flow-through capillary, in 6 consecutive 10 min exposures. Ovotransferrin (Ovo) and bovine serum albumin (BSA) solutions with BS^3^ and FA were measured in a flow-through capillary, in 6 consecutive 10 min exposures. The 2D scattering patterns were azimuthally integrated, using the FIT2D program.^57^ The scattering intensity, *I*, as a function of the magnitude of the momentum transfer vector, *q*, was plotted and analyzed using our in-house software X+ ^58–60^ and D+.^31–33,61,63^ The azimuthally integrated scattering intensities were scaled to absolute units as explained.^62^

### Models

Using our in-house analysis software, D+,^31,32,60^ we generated the expected solution scattering curves based on atomic models. For all the models computed by D+, we included the contributions of the monomer and dimer hydration layers. The software accounted for the contribution of voxels surrounding the protein with the following parameters: a solvent voxel size of 0.2 nm, a solvent probe radius of 0.14 nm, a solvation thickness of 0.2 nm, and a hydration layer electron density of 364 e^–^/nm^3^.^64^

### Mass Spectrometry

Trypsin peptide digest was prepared as described.^35^ The samples were analyzed by a 120 min 0–40% acetonitrile gradient on a liquid chromatography system coupled to a Q-Exactive Plus mass spectrometer (Thermo). To keep the FA cross-links intact we kept the temperature of the sample below 40 °C throughout all the preparation stages (alkylation, digestion, desalting, and in the analytical column of the LC). The RAW data file format obtained from the mass spectrometer was converted to MGF format by Proteome Discoverer (Thermo), and analyzed for cross-links as described.^18^ The method parameters of the MS analyses were: data-dependent acquisition; full MS resolution 70,000; MS1 AGC target 1 × 10^6^; MS1Maximum IT 200 ms; scan range between 450 and 1800; dd-MS/MS resolution 35,000; MS/MS AGC target 2 × 10^5^; MS2 maximum IT 300 ms; loop count Top 12; isolation window 1.1; fixed first mass 130; MS2 minimum AGC target 800; charge exclusion: unassigned, 1, 2, 3, 8, >8; peptide match—off; exclude isotope—on; dynamic exclusion 45 s.

## Results

### Microtubule Structure at Different Exposure Times and pH Values

Microtubules (MTs) are hollow cylindrical protein polymers composed of laterally associated protofilaments, which are linear chains of αβ-tubulin heterodimers. Inside eukaryotic cells, MTs have 13 protofilaments. *In vitro*, the protofilament number in MTs assembled from purified tubulin typically ranges between 12 and 15, where 14 is most common. The protofilament number influences the overall diameter and mechanical stability of MTs and is an important factor when interpreting SAXS data from MTs.^23^

*In vitro* MT structure determination is typically performed at a pH of 6.9 and involves short exposure times to radiation.^24–26^ In contrast, cross-linking reactions in XL-MS studies typically require a pH of 7.0 or higher and incubation times of tens of minutes. We therefore first examined by SAXS the effects of pH and exposure time on the MT structure (Figure 1). MT polymerization at 36 °C was induced by adding excess GTP and measured in our in-house high-resolution SAXS setup using a flow-through capillary.^27^ All the MT measurements employed a supernatant background subtraction step,^23^ which was required because MTs coexist with smaller tubulin assemblies, such as single rings, ring fragments, and other small aggregates, which do not precipitate during centrifugation (Figure 1A).^28–30^ By subtracting the SAXS signal of these small assemblies, existing in the supernatant following centrifugation of the sample, from the signal of the entire sample before the centrifugation was applied, we could isolate the scattering signal from the MTs more accurately.

**Figure 1:**
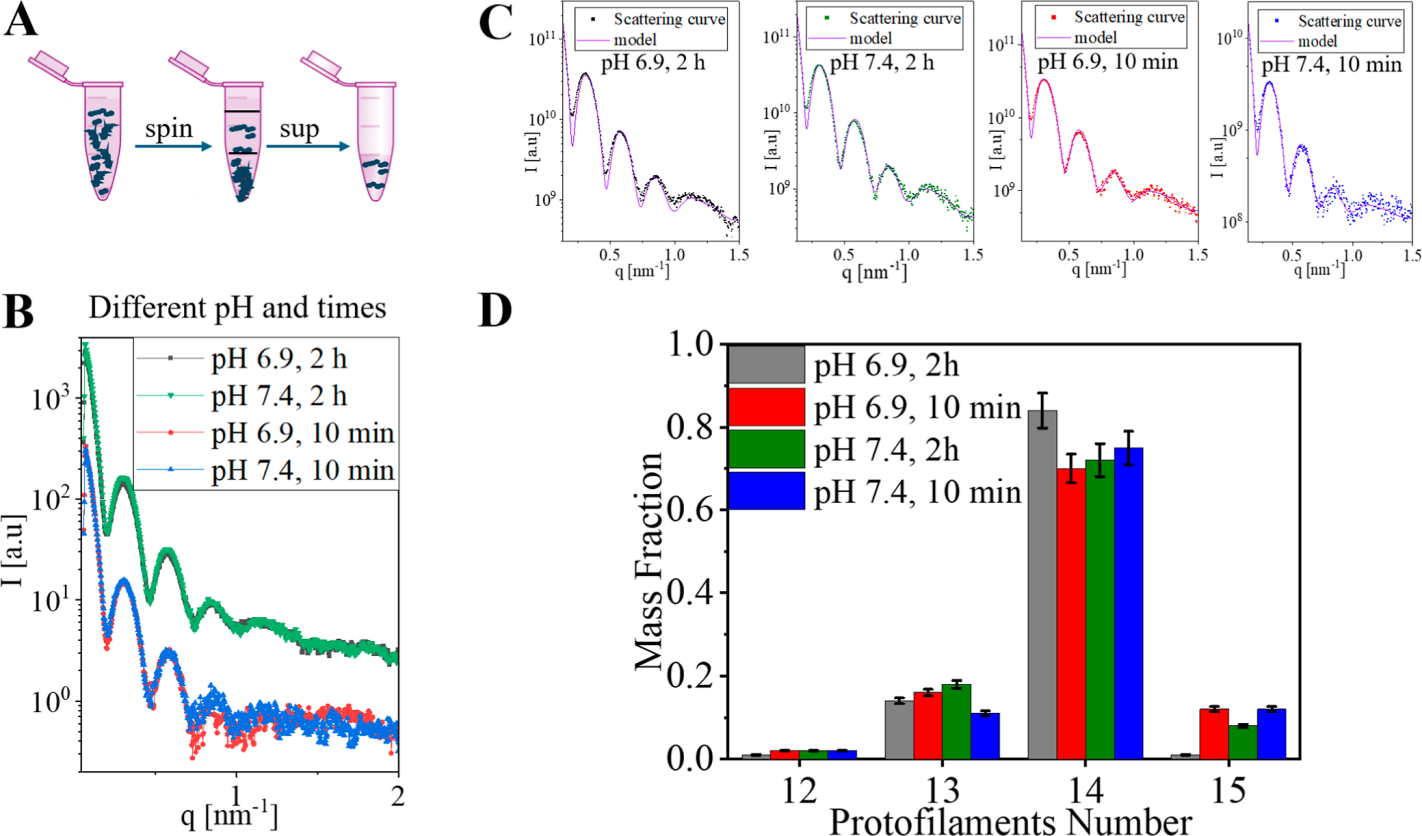
Samll-angle X-ray scattering (SAXS) measurement and modeling of the microtubule (MT) structure. (A) Schematic of background subtraction, in which the SAXS signal of free tubulin dimers and small tubulin assemblies (right tube) is subtracted from the signal of the entire sample (left tube). (B–D) Effects of pH value and measurement exposure time on MT structure. (B) Azimuthally integrated supernatant-subtracted solution SAXS intensity curves are plotted as a function of the magnitude of the scattering vector, q. Top curve: SAXS measurement exposure time of 2 h at pH 6.9 (gray) and 7.4 (green). Bottom curves: 10 min exposure at pH 6.9 (red) and 7.4 (blue). (C) Model fit (purple curves) to the SAXS measurements, computed from the best-fitted non-negative linear combination of atomic models of MT (based on PDB_ID: 3J6F^24^), containing between 12 and 15 protofilaments (Figure S1). The final models (the linear combinations) were convolved with an estimated Gaussian instrument resolution function with a standard deviation of 0.03 nm^–1^. (D) The mass fraction of each protofilament number in the models that best fit the data in (C).

The two pH values (6.9 and 7.4) and two exposure times (10 min and 2 h) had a negligible effect on the SAXS signals (Figure 1B). This encouraging result implies that under pH conditions that are compatible with chemical cross-linking the MT structure remained unchanged within the resolution of our SAXS measurements. To analyze any possible changes in the distribution of MT protofilament number, we computed the theoretical scattering curves from models of MTs, containing between 12 and 15 protofilaments (Figure S1), using the D+ software.^23,31–33^ The best fit of a non-negative linear combination of these MT models to the SAXS curves (Figure 1C) gave similar distributions of MT protofilament number for all pH values and measurement exposure times (Figure 1D). Overall, the best-fitting distribution assigned roughly 2% of the tubulin mass fraction to 12 protofilaments, 15% to 13 protofilaments, 75% to 14 protofilaments, and 8% to 15 protofilaments, with a relative error of about 5%. These findings are consistent with those reported in our previous study.^23^

### Effects of Cross-Linking on MT Structure

We added 10 mM BS^3^, 0.1 wt % FA, or 1 wt % FA to MT solution at pH 7.4, and measured the SAXS intensity curves at increasing incubation times (Figure 2). Cross-linking with BS^3^ had very little effect on the SAXS signal, even after 1 h incubation (Figure 2A). In contrast, cross-linking with 1 wt % FA rapidly degraded the SAXS signal within 10 min or less (Figure 2C). Cross-linking with a 10-fold lower concentration of FA changed the SAXS signal only after a much longer incubation (Figure 2B). An overlay of the SAXS signals after 20 min of incubation with the different reagents highlights the contrast between BS^3^ and FA at low and high concentrations (Figure 2D). We note that the BS^3^ concentration used here (10 mM) is higher than the typical concentrations used in many XL-MS studies.^34–38^ The high concentration of reagent was required to match the high concentration of protein in the MT solution (90 μM of tubulin dimer) and the final reagent-to-tubulin molar excess was 110.

**Figure 2:**
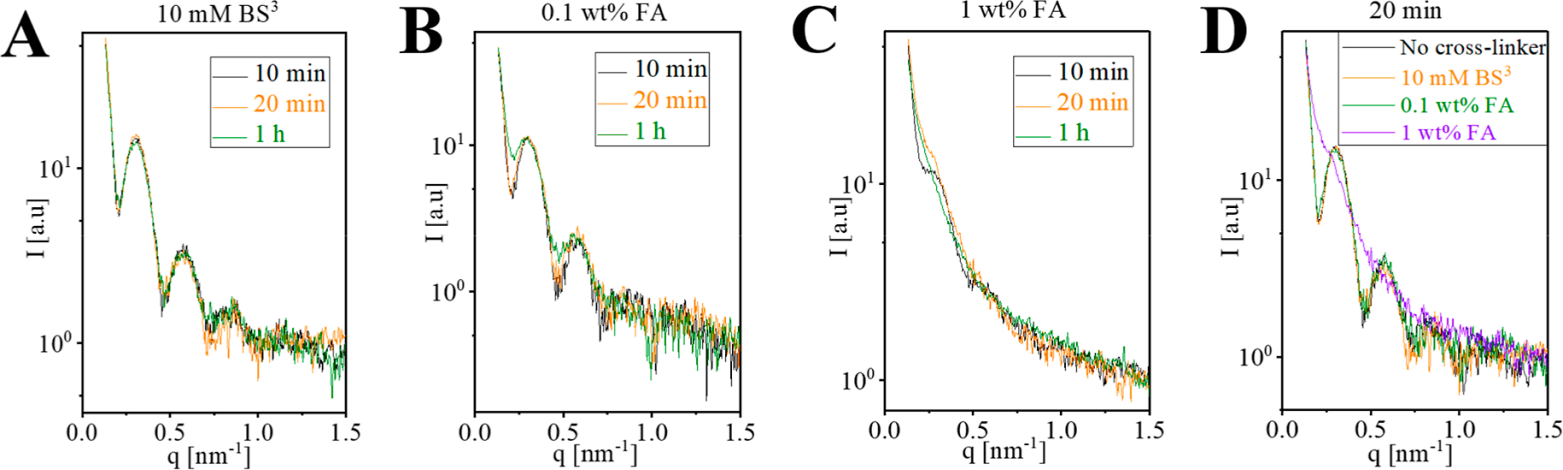
Effects of bis­[sulfosuccinimidyl] suberate (BS^3^) and formaldehyde (FA) cross-linking on the SAXS curves of MT. All measurements were performed using MT solutions at a pH of 7.4. The corresponding supernatant background was subtracted from each measurement. (A–C) Three different incubation times with either 10 mM BS^3^, 0.1 wt % FA, or 1 wt % FA. (D) Overlay of the SAXS curves following 20 min incubation with the indicated cross-linking reagents. The analysis of the data is shown in Figure 3.

The minor structural effect of cross-linking with BS^3^ or low-concentration FA was further demonstrated by fitting structural models to the SAXS data after 20 min of incubation with the reagents (Figure 3). Good fit to the data of a non-negative linear combination of canonical MT models containing between 12 and 15 protofilaments^23^ was found for both cross-linked and non-cross-linked samples (Figure 3A). The protofilament number distribution was likewise indistinguishable (within error) between the samples (Figure 3B). These analyses indicate that cross-linking with 10 mM BS^3^ or 0.1 wt % FA had little effect on the MT structure, within the measurement resolution limits. A fit based on Porod’s law was applied to the scattering data for 1 wt % FA at low *q*-values. The data were well described by a *q* ^–4^ dependence, consistent with Porod’s law, indicating the presence of large, compact aggregates with well-defined spherical interfaces (Figure 3C).

**Figure 3:**
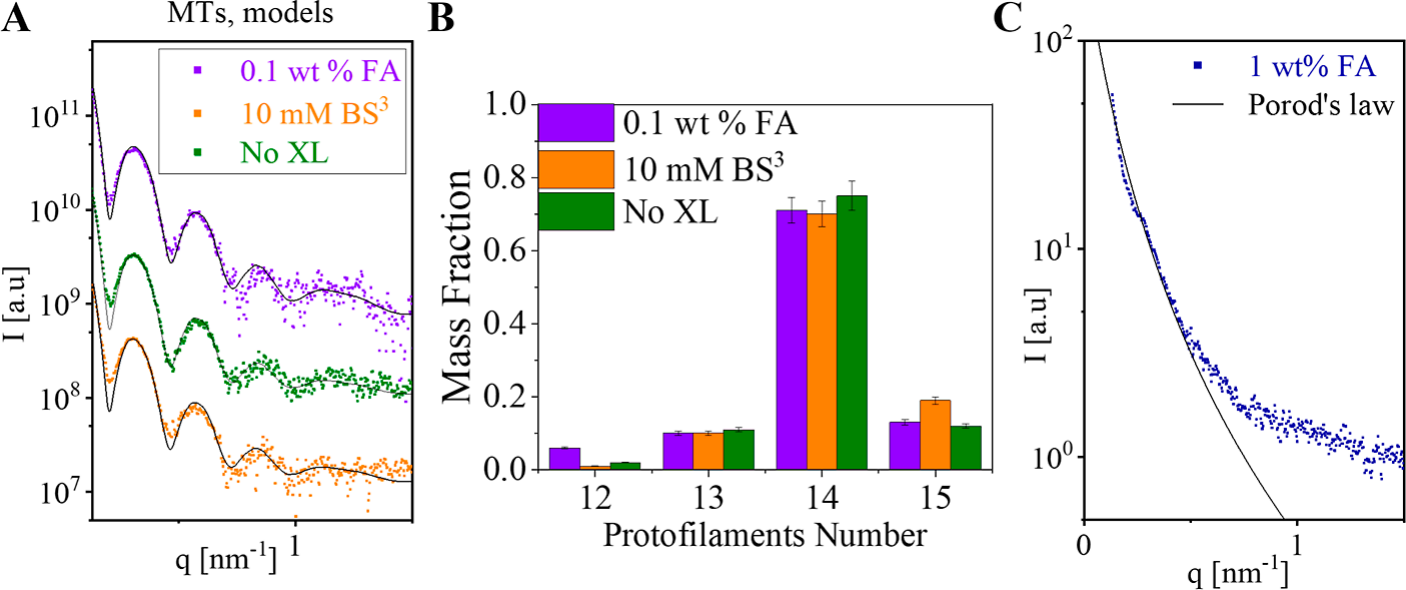
Analysis of cross-linked MTs. (A) The MT models (black curves) that best fitted the supernatant subtracted SAXS measurements (symbols) from MT solutions after 20 min of incubation with the indicated cross-linking reagents. The models were computed as explained in Figures 1 and S1. (B) The mass fraction of tubulin as a function of MT protofilament number in the models that best fit the data in (A). (C) Porod’s law was applied to the 1 wt % formaldehyde (FA) scattering curve, yielding a good fit at low *q*-values with a Porod exponent of -4.

In parallel to the SAXS measurements, the cross-linked samples were subjected to XL-MS analyses to identify the cross-linked sites. The addition of 10 mM BS^3^ yielded a total of 46 cross-links across three experimental replicates, with 17 cross-links consistently identified in all three repeats (Figure 4A and Table S1). Each cross-link is either from a single tubulin unit (intra) or bridging two adjacent tubulin units (inter) within the context of the MT tubule. This ambiguity can largely be resolved based on cross-link mapping onto the MT structure. To that end, an atomic model of the full MT tubule, including the additional interface that closes the tubule (seam), was reconstructed based on the coordinates and helical parameters from PDB_ID 3J6F.^24^ All the possible mappings of each cross-link onto the MT tubule were exhaustively tested, and the cross-link was classified as intra/inter/seam based on the mapping with the shortest span. Most of the identified cross-links were consistent with the atomic MT structure, falling within the accepted distance limit of 30 Å between Cα atoms^21^ (Figure 4C). However, 23% of the cross-links exceeded this distance limit. We hypothesized that these additional cross-links originated from alternative tubulin assemblies coexisting in the solution, for which high resolution atomic structures are not available.^23,28,39^ To isolate nonmicrotubule cross-links, we adapted the SAXS background subtraction protocol to the XL-MS pipeline. The MT original solution, which contained both MT and other (non-MT) tubulin assemblies, including tubulin single rings, ring fractions, and other oligomers, was centrifuged to precipitate the heavier MT fraction. XL-MS analysis of the supernatant identified 33 cross-links. Comparing these to the 17 common cross-links from the entire sample revealed a subset of 9 cross-links associated exclusively with MTs (Figure 4B). All 9 MT-exclusive cross-links mapped to the MT structure with distances shorter than 25 Å, supporting our hypothesis.

**Figure 4:**
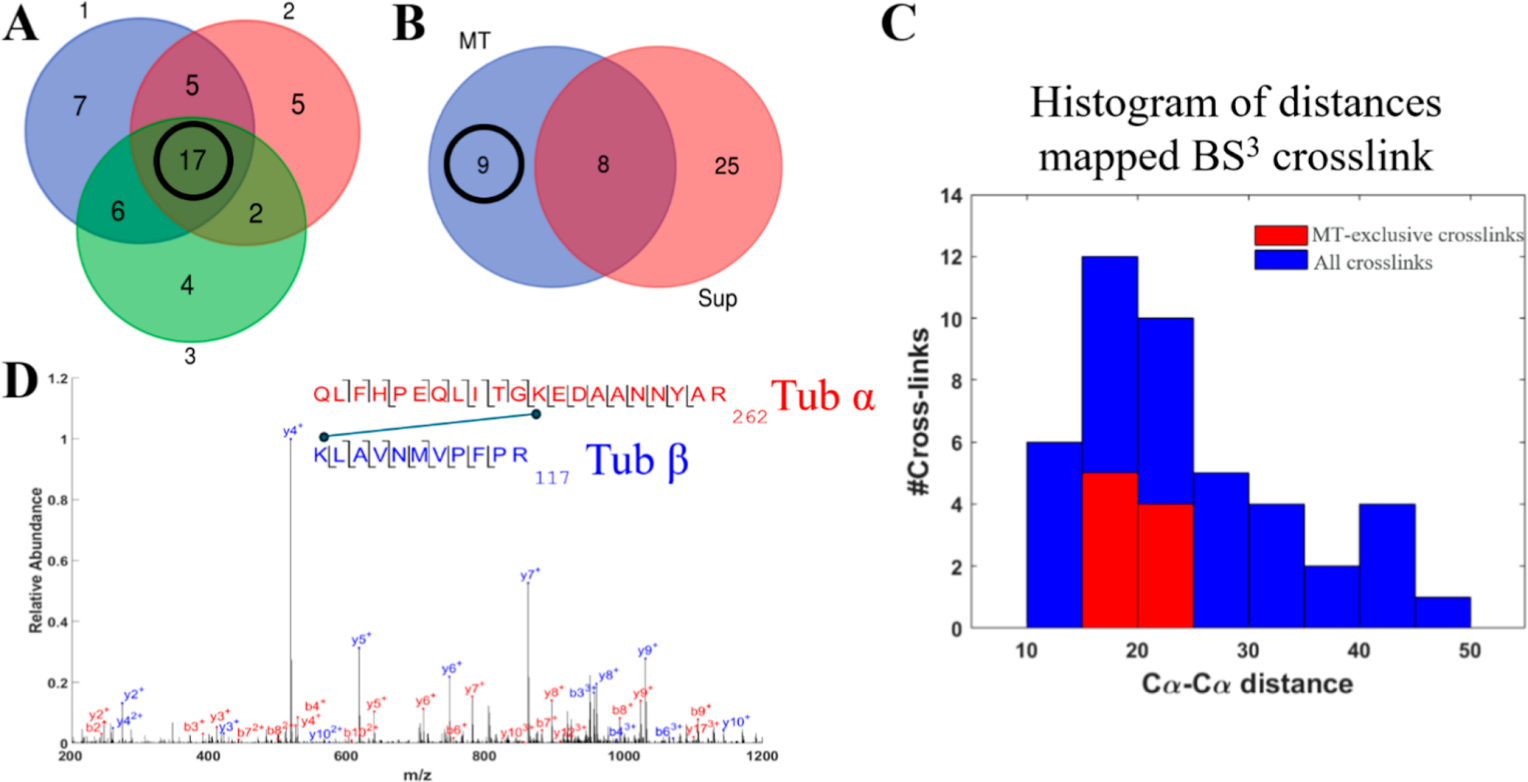
Identifying MT-exclusive BS^3^ cross-links. (A) Venn diagram of the cross-links identified from MT solutions. Seventeen cross-links were identified in all three repeats. (B) Comparison of the 17 repeating cross-links from the MT solutions (blue) with those identified from the supernatant (red). Nine cross-links (out of the 17) are associated exclusively with MTs. (C) Histograms of the distances spanned by the cross-links in the solved MT atomic structure. The cross-links from all three repeats are in blue, and the 9 cross-links exclusive to MTs are overlaid in red. (D) Annotated MS/MS spectrum of a cross-link between two peptides from α-tubulin (red) and β-tubulin (blue).

The same XL-MS analysis pipeline for FA cross-linking revealed a similar pattern of consistency with the atomic structure of MTs (Figure S2). Cross-linking with 0.1 wt % FA identified 8 common cross-links in two experimental repeats, which were all consistent with the MT atomic structure. Cross-linking with 1 wt % FA gave a much higher yield comprising 56 common cross-links in two experimental repeats. Yet, the 1 wt % FA cross-links were less consistent with the atomic MT structure than the BS^3^ cross-links, with 30% of all the cross-links spanning a distance longer than 30 Å. Nonetheless, as for the BS^3^ cross-linking, a subset of ten MT-exclusive cross-links was fully consistent with the MT structure.

### Effects of Cross-Linking on the Tubulin Dimer

We used the same experimental approach to study the effects of cross-linking on solutions that are rich in tubulin dimer, the repeating subunit of MT filaments. A solution of 10 mg/mL tubulin was kept at 4 °C to prevent MT assembly and subjected to cross-linking by either 10 mM BS^3^, 0.1 wt %, or 1 wt % FA. The scattering of the tubulin was measured using our in-house high-resolution solution SAXS setup at different incubation times (Figure 5). As in the case of the MTs, cross-linking with 10 mM BS^3^ had an unnoticeable effect on the SAXS signal even at the longest incubation time of 1 h (Figure 5B). On the other hand, cross-linking with 1 wt % FA significantly increased the intensity at the low-*q* range (*q* < 0.5 nm^–1^), within 10 min incubation (Figure 5D), indicating the rapid accumulation of tubulin aggregates. The low-*q* region provides insight into the larger-scale structural organization of the protein (in fact, at *q* = 0, the intensity is proportional to the average mass of the assemblies), again consistent with Porod’s law (Figure S3). Cross-linking with 0.1 wt % FA also led to a low-*q* intensity increase (Figure 5C) but became noticeable only after more than 20 min incubation (Figure 5E).

**Figure 5:**
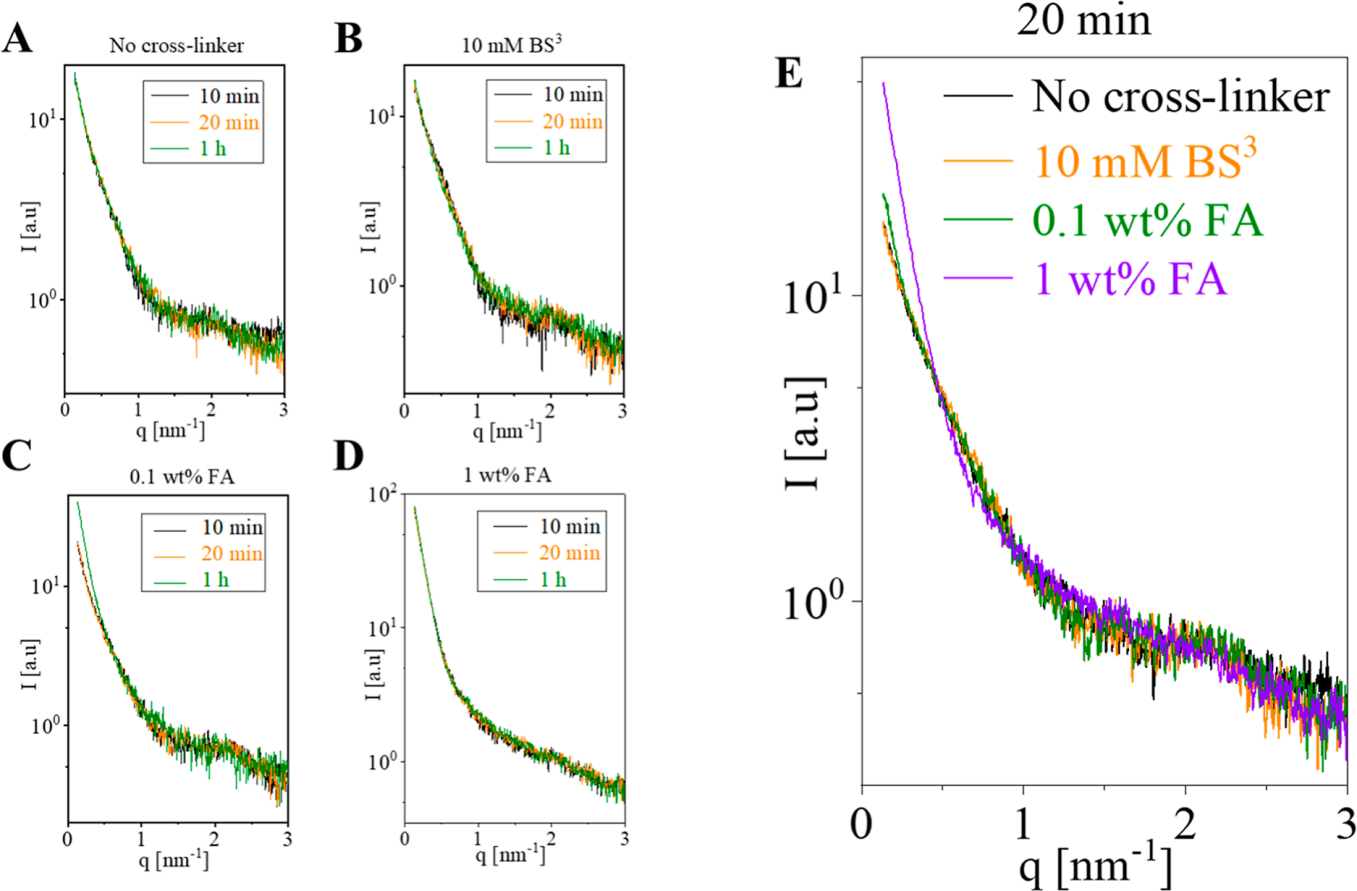
Effects of BS^3^ and FA cross-linking on tubulin. Background-subtracted SAXS measurements of tubulin solutions at a pH of 7.4 and at 4 °C (the low temperature inhibits MT polymerization). (A–D) SAXS curves at three incubation times with either no reagent, 10 mM BS^3^, 0.1 wt % FA, or 1 wt % FA. (E) Overlay of the SAXS curves following 20 min incubation with the different cross-linking reagents.

Earlier studies have shown that larger tubulin complexes (tubulin single rings, ring-fragments, and other tubulin oligomers) coexist in equilibrium with tubulin dimers.^28,40,41^ Therefore, no attempt was made to fit an atomic model of the tubulin dimer to these data. Indeed, XL-MS analysis of the cross-linked tubulin dimer provided further evidence for these larger structures by identifying cross-links that are incompatible with the solved tubulin atomic structure (PDB ID 1JFF^42^). We identified a total of 58 BS^3^ cross-links in two experimental repeats, 27 of which appeared in both repeats (Figure S4 and Table S2). Yet, owing to the presence of tubulin aggregates, more than 40% of these cross-links spanned distances longer than the accepted 30 Å limit.

### Effects of Cross-Linking on Ovotransferrin and Bovine Serum Albumin

To further test the generality of the previous observations, we measured two additional globular proteins, ovotransferrin (OVO) and bovine serum albumin (BSA). First, we cross-linked 8 mg/mL OVO solution in BRB80 buffer at room temperature by either 1 mM BS^3^, 0.1 wt %, or 1 wt % FA, and prepared a non-cross-linked control. We then performed SAXS measurements using our in-house SAXS setup at two incubation times (Figure 6). Similarly to the tubulin dimers, cross-linking with BS^3^ or 0.1 wt % FA had no noticeable effect on the SAXS curves, whereas cross-linking with 1 wt % FA increased the low-*q* intensity, suggesting aggregate formations (Figure 6E). The scattering data at low *q*-values were well described by a *q* ^–4^ fit, consistent with Porod’s law, suggesting the presence of large, compact aggregates with sharp interfaces. The effect of 1 wt % FA cross-linking was also rapid and occurred within 10 min of incubation with the reagent. In parallel to the SAXS measurements, XL-MS analyses identified an average of 44, 19, and 52 cross-links formed by 1 mM BS^3^, 0.1 wt %, and 1 wt % FA, respectively (Figure S5 and Table S3). In agreement with the SAXS results, mapping of the BS^3^ and 0.1 wt % FA cross-links onto the crystallographic structure of OVO (PDB ID 1aiv^43^) found nearly all of the cross-links to be within the expected limits. The 1 wt % FA cross-links were generally also in agreement with the atomic structure, except for ∼10% which spanned significantly longer distances (Figure S5L).

**Figure 6:**
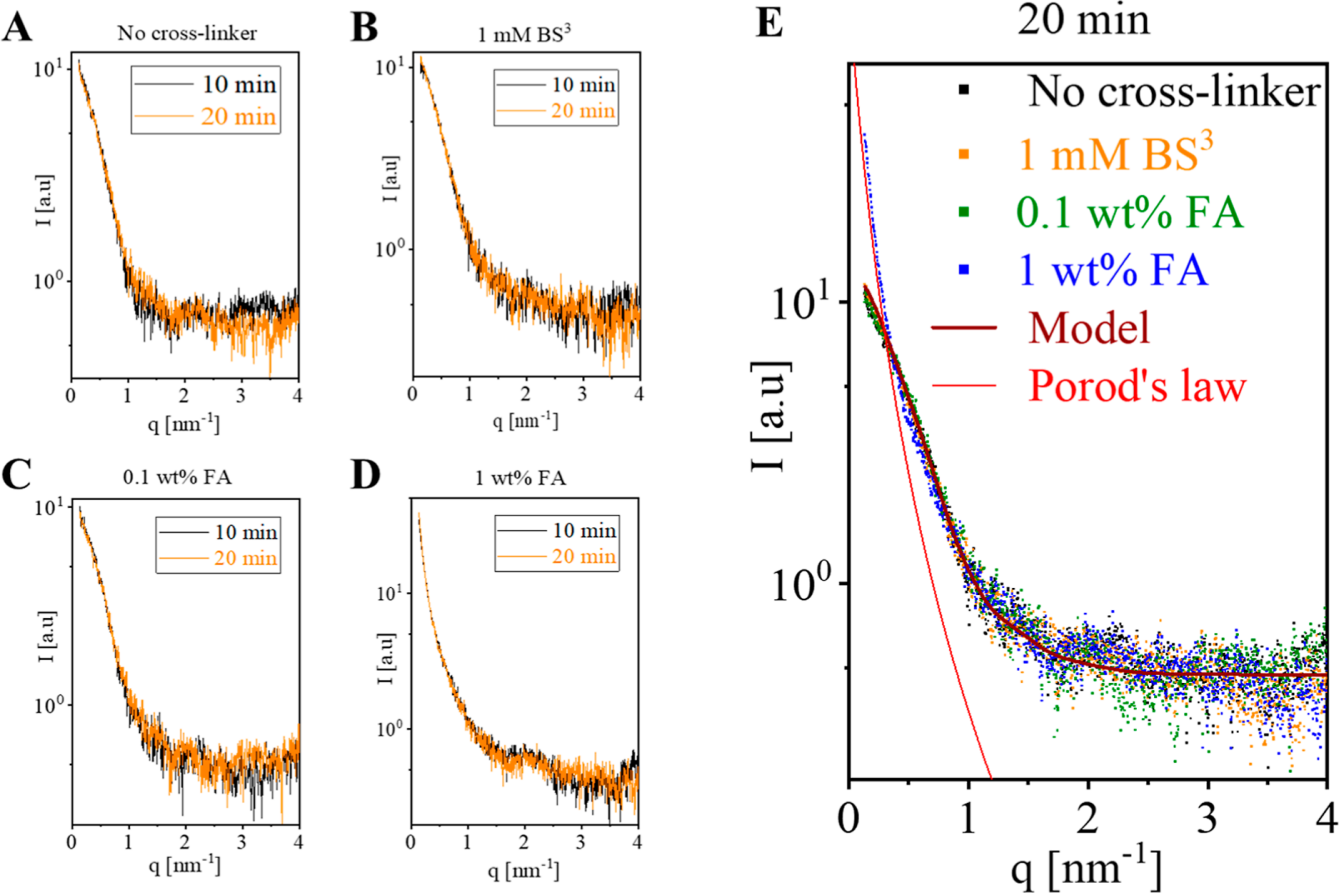
Effects of BS^3^ and FA cross-linking on ovotransferrin (OVO). (A–D) Background-subtracted SAXS curves at two different incubation times with either no reagent (A), 1 mM BS^3^ (B), 0.1 wt % FA (C) or 1 wt % FA (D). (E) Overlay of the SAXS curves following 20 min incubation with the indicated cross-linking reagents. The best-fitted computed model (using D+) based on a linear combination of the atomic structure of OVO monomer and dimer is overlaid (solid brown curve). The computed monomeric and dimeric models are shown in Figure S6. For the low q values of 1 wt % FA, a fit to Porod’s law was performed (red).

The availability of a crystallographic structure for OVO allowed us to fit an atomic model to the SAXS data. A preliminary result was computed by D+ from a hydrated model of the crystallographic structure (see Section Materials and Methods). Yet, this monomeric model alone did not fit the data sufficiently well. We therefore added a dimer model of two OVO monomers separated by 9 nm, which is the average diameter of the monomer. The best fit to the data was obtained when the mass fractions of monomeric and dimeric OVO were 82% and 18%, respectively (Figure 6E).

SAXS measurements of cross-linked bovine serum albumin (BSA) similarly presented the same effects of 1 wt % FA cross-linking observed for OVO (Figure S7). In this case, Porod’s law did not adequately fit the scattering curve for the 0.1 wt % FA dispersion. A monomeric model alone was again insufficient for a satisfactory fit to the SAXS data. We therefore used PDB IDs 4f5u^44^ and 3v03 ^45^ for computing the monomer and the dimer models, respectively (Figure S8). Using the default solvation parameters, the best fit to the data was obtained when the mass fractions of monomeric and dimeric BSA monomers were 71% and 29%, respectively. The higher fraction of dimers in the model is consistent with the known tendency of BSA to form oligomers in solution.^46^ XL-MS analyses of BSA identified an average of 120, 11, and 21 cross-links from 1 mM BS^3^, 0.1 wt %, or 1 wt % FA, respectively (Figure S8 and Table S4). About ∼15% of the BS^3^ cross-links were inconsistent with the atomic structure of the BSA monomer, most likely owing to the abundant oligomeric interfaces (Figure S9).

## Discussion and Conclusions

This study relied on solution SAXS to directly track the structural changes in proteins during chemical cross-linking. The wide applicability of the SAXS approach enabled testing several cross-linking reagents and concentrations, as well as a range of protein systems greatly varying in size. It also allowed us to follow the course of the structural changes by measuring the same sample at successive time points. The SAXS data revealed structural changes associated with the aggregation of the proteins leading to increased low-*q* intensity (Figures 3 and 6 and S3), and the loss of the well-defined self-assembled MT structures leading to smearing of the oscillatory scattering curves.

Overall, a consistent picture arises across all the measured protein systems. Cross-linking with BS^3^ had a negligible effect on protein structures, even at prolonged incubation times. Within the resolution limits of the SAXS data, samples cross-linked with BS^3^ were essentially indistinguishable from control samples without cross-linking. In contrast, cross-linking with 1 wt % FA led to drastic structural changes at time scales shorter than 10 min. These changes appear to be severe and may compromise the biological relevance of the cross-linked structure. Cross-linking with 0.1 wt % FA led to intermediate effects in which some structural changes were observed but only at prolonged incubation times.

The SAXS measurements of the BS^3^-treated samples agree with NMR measurements of similarly treated globular proteins,^22^ showing only mild structural perturbations. BS^3^ is a member of a large class of cross-linking reagents, based on NHS ester chemistry, and mainly reacts with lysine side chains. The common view in the XL-MS field is that reagents of this class generally preserve protein structure as long as their working concentrations are not excessive. The SAXS and NMR results empirically justify this view and strongly support using these reagents. Indeed, NHS ester reagents, such as BS^3^, DSSO,^47^ and DSBU^48^ are by far the most common in XL-MS studies. Their use is also increasing in related applications, such as the stabilization of protein complexes for cryoEM.^49–51^

The severe structural effects of FA cross-linking may appear contradictory to common experience. FA is widely used for chemical fixation of cells and tissues, and follow-up imaging by light and electron microscopy generally shows that the cellular substructures are preserved.^52^ Furthermore, our own experience with in-cell FA cross-linking found that the identified cross-links were in good agreement with known atomic structures.^18,35,53^ The most likely explanation to reconcile this discrepancy is the difference between in-cell and solution cross-linking. Macromolecules within the cell encounter a tight environment that is fortified with large cytoskeletal and organellar structures. In the crowded cellular context, molecules are restricted and fewer structural changes are expected following cross-linking. Any conclusion from this study is therefore relevant mainly to in-solution applications. We also note that even in solution, more than 80% of the FA cross-links are still in agreement with known atomic structures. Therefore, the FA cross-link sets may be used, but with more scrutiny.

In conclusion, our results support the following recommendations for in-solution structural studies aided by cross-linking. The use of NHS ester chemistry should be preferred to aldehyde-based chemistry when possible. When FA cross-linking is used, lowering the working FA concentration is beneficial. Finally, solution X-ray scattering analysis can verify the integrity of the structure following cross-linking, and can be applied with relative convenience and speed.

## Supplementary Material











## Notes

Compilation of all microtubule cross-links (Table S1) (XLSX)
Compilation of all tubulin cross-links (Table S2) (XLSX)
Compilation of all Ovotransferin cross-links (Table S3) (XLSX)
Computed scattering curves from microtubule models with different protofilament numbers (Figure S1) XL-MS analysis of MT solution (Figure S2) A fit of Porod’s law for tubulin (Figure S3) XL-MS analysis of tubulin solution (Figure S4) XL-MS analysis of Ovotransferin solution (Figure S5) Computed scattering curves from Ovotransferin models (Figure S6) SAXS scattering data for BSA (Figure S7) Computed scattering curves from BSA models (Figure S8) XL-MS analysis of BSA solution (Figure S9) (PDF)
Compilation of all BSA cross-links (Table S4) (XLSX)
