# Supplementary material for: Effects of Chemical Cross-Linking on the Structure of Proteins and Protein Assemblies
Source: Anal Chem. 2025 Jul 8;97(28):15104–12. doi: 10.1021/acs.analchem.5c01092 (PMC12291049; doi:10.1021/acs.analchem.5c01092)
Supplement: Supplementary file 4 [file ac5c01092_si_004.pdf]

Supplementary Information

Effects of chemical crosslinking on the structure of proteins and protein assemblies

Tamar Tayri-Wilk[1], Nir Kalisman[2,3,*], Uri Raviv[1,2,*]

[1] Institute of Chemistry, The Hebrew University of Jerusalem, Edmond J. Safra Campus, Givat Ram, 9190401, Jerusalem, Israel.

[2] Center for Nanoscience and Nanotechnology, The Hebrew University of Jerusalem, Edmond J. Safra Campus, Givat Ram, 9190401, Jerusalem, Israel.

[3] Institute of Life Sciences, The Hebrew University of Jerusalem, Edmond J. Safra Campus, Givat Ram, 9190401, Jerusalem, Israel.

*Correspondence: nirka@mail.huji.ac.il (N.K.), uri.raviv@mail.huji.ac.il (U.R.)

Supplementary Information includes:

Figure S1. Computed scattering curves, microtubule, different protofilament numbers.
Figure S2. XL-MS analysis of MT solution.
Figure S3. A fit of Porod’s law, tubulin.
Figure S4. XL-MS analysis of tubulin solution.
Figure S5. XL-MS analysis of Ovotransferin solution.
Figure S6. Computed scattering curves, Ovotranferin, monomer and dimer.
Figure S7. SAXS scattering data for BSA.
Figure S8. Computed scattering curves, BSA, monomer and dimer.
Figure S9. XL-MS analysis of BSA solution.
Table S1. Compilation of all microtubule crosslinks.
Table S2. Compilation of all tubulin crosslinks
Table S3. Compilation of all ovotransferin crosslinks
Table S4. Compilation of all BSA crosslinks

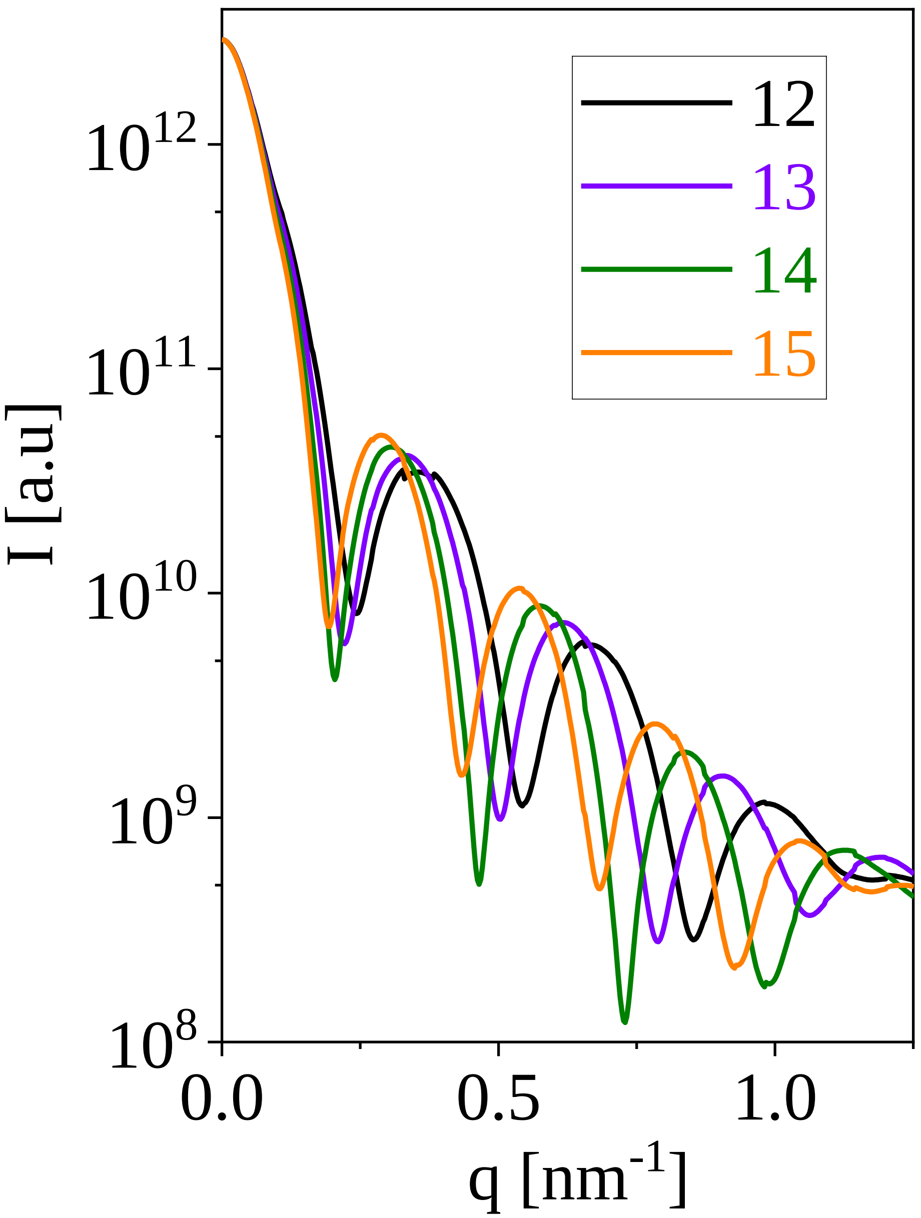

Figure S1. The computed scattering curves from microtubule models containing between 12 and 15 protofilaments, used for analyzing the distribution of protofilament number in Figures 1 and 3. In each model, the tubulin-dimer basic subunits were arranged in 3-start left-handed helical lattices with a pitch of 12.2 nm and radii to the geometric center of the dimer atomic coordinates, R, of 10.2, 11.05, 11.9, and 12.75 nm, corresponding to 12, 13, 14, and 15 protofilaments, respectively. The models had 11 dimers in each protofilament (i.e. the protofilaments were 91 nm long).

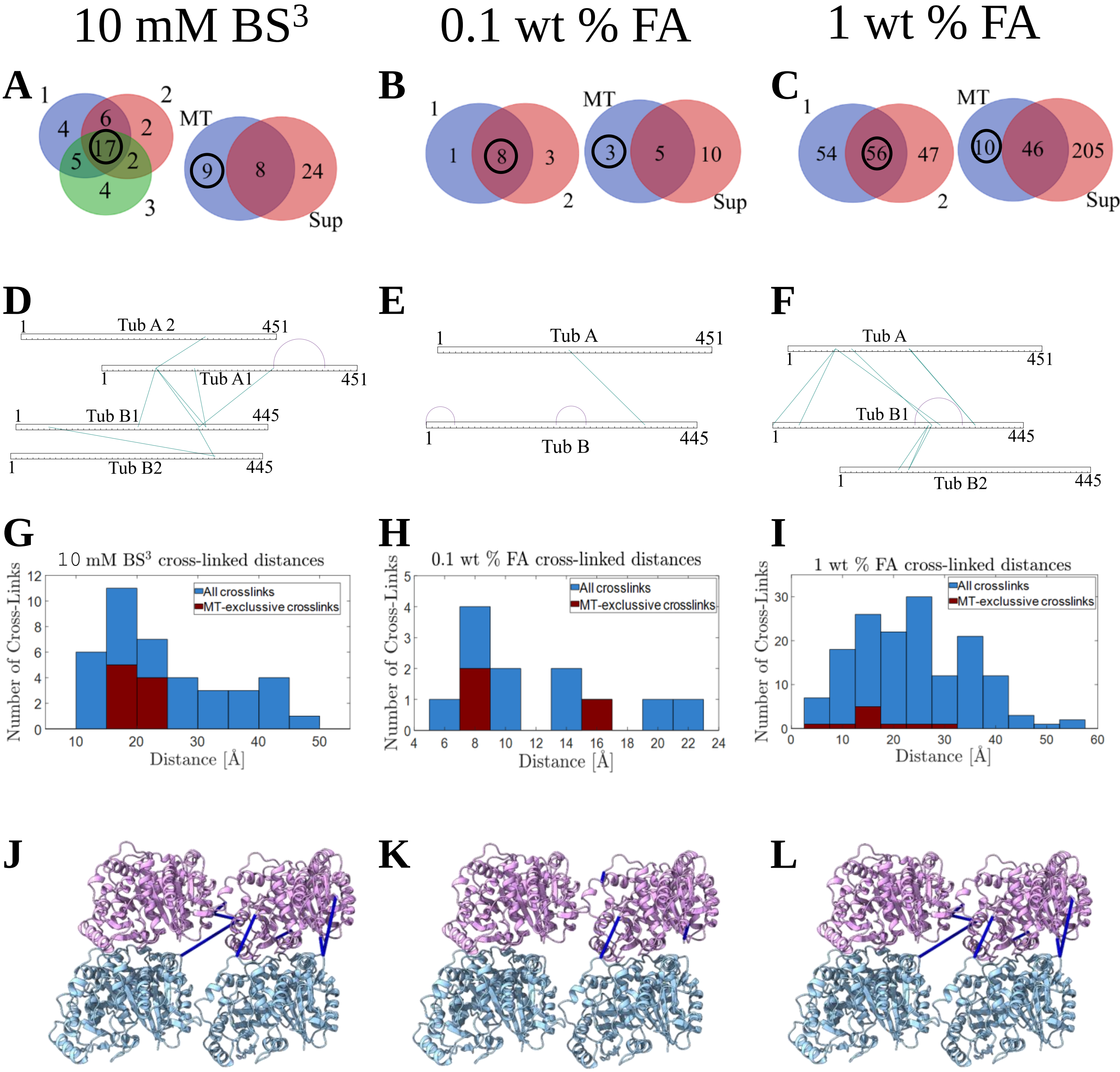

<u>Figure S2.</u> XL-MS analysis of MT solution. **(A-C)** Venn diagrams showing cross-links of 10 mM BS^3 **(A),** 0.1 wt% FA **(B)**, and 1 wt% FA **(C)**. For each crosslinking condition, the left diagram details the yields of experimental repeats and the right diagram compares the common crosslinks in all repeats (MT, blue) with the crosslinks identified in the supernatant of the background subtraction (Sup, red). The circled numbers in each right diagram indicate the MT-exclusive crosslinks. **(D-F)** Connectivity maps of the MT-exclusive crosslinks. Two copies of alpha and beta tubulin sequences are presented to separate intra- and inter-subunit crosslinks. **(G-I)** Histograms of the distances that the common (blue) and the MT-exclusive (red) crosslinks span on the atomic structure of the microtubule. **(J-L)** Mapping of the MT-exclusive crosslinks on a section of the atomic structure of the microtubule tube. Alpha and beta tubulin subunits are colored pink and blue, respectively.

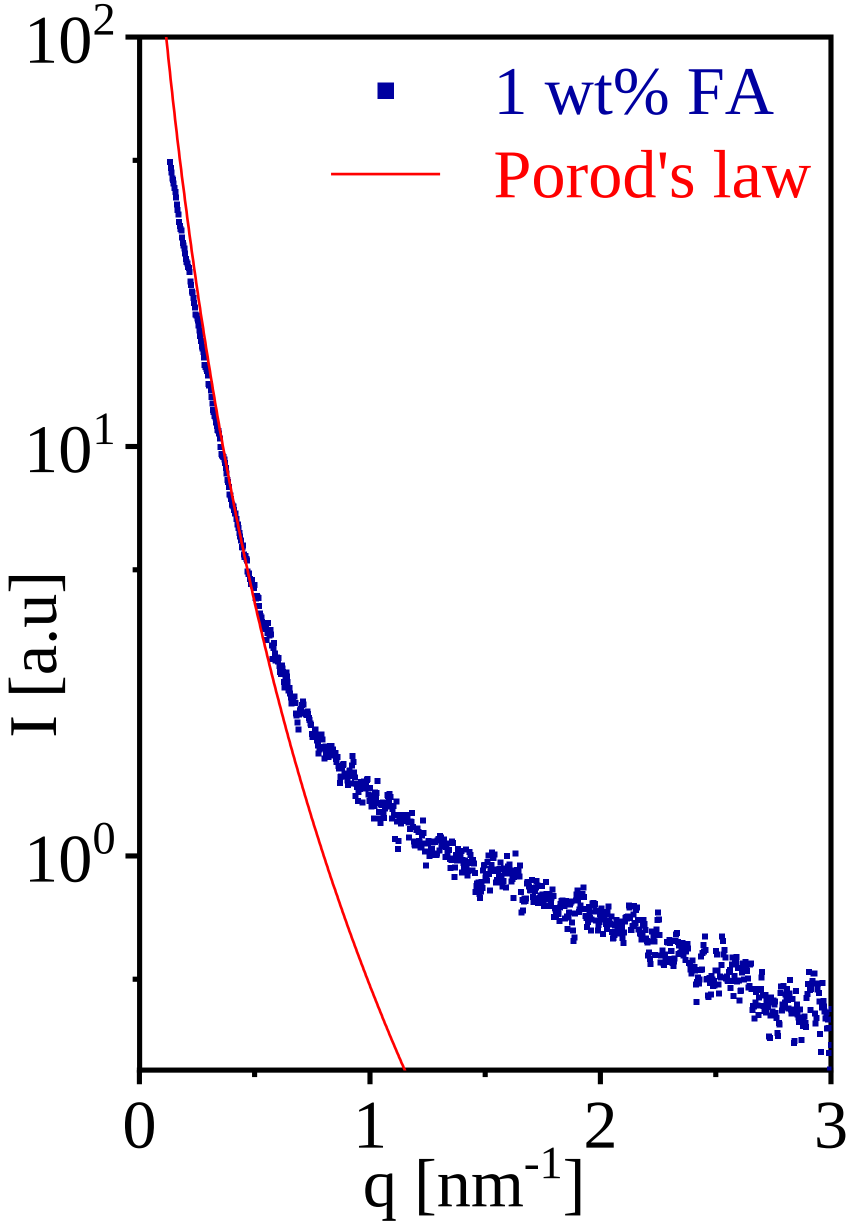

Figure S3. A fit of Porod’s law (red) for the SAXS curve of Tubulin crosslinked with 1 wt% FA (blue symbols).

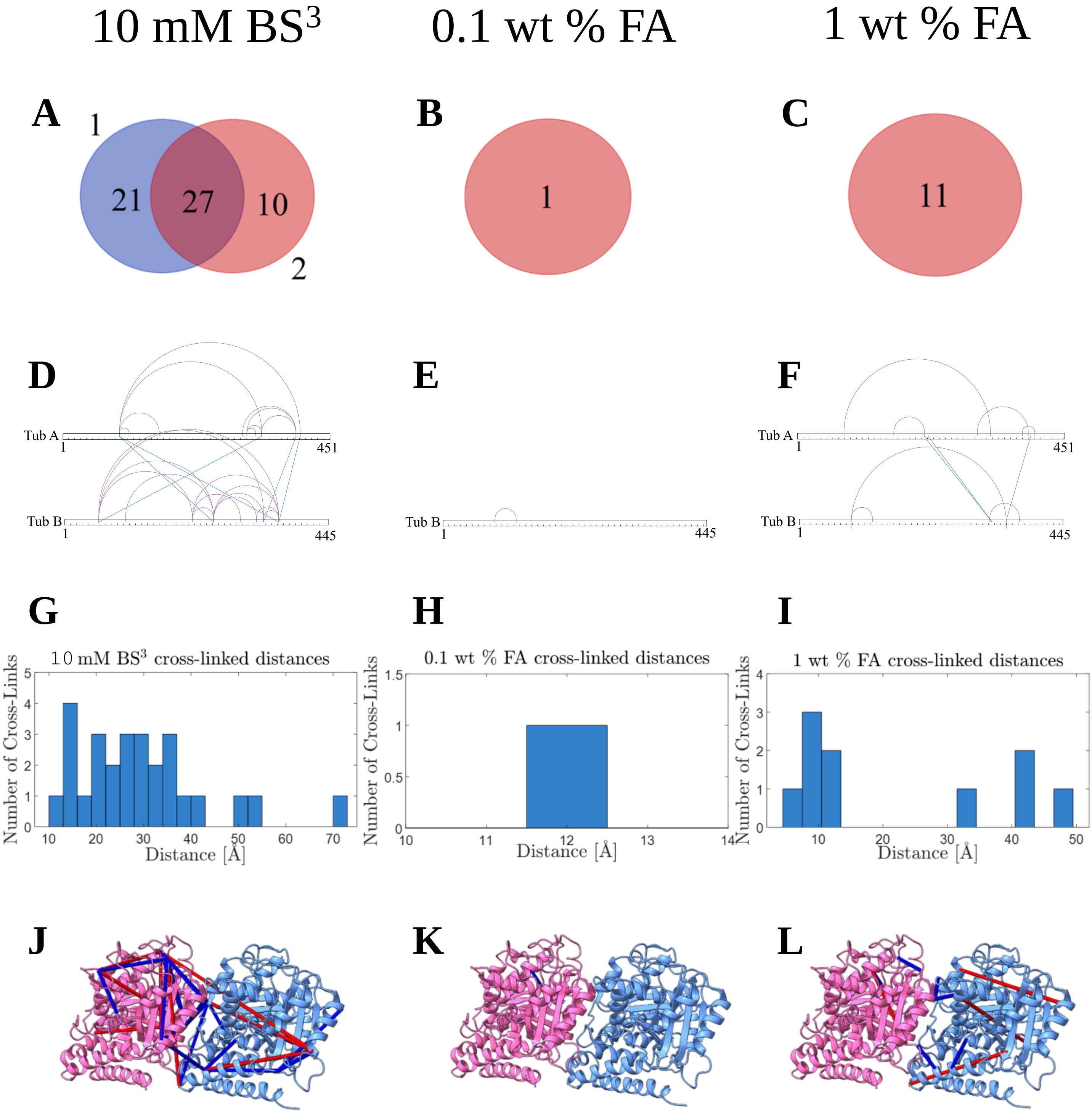

Figure S4. XL-MS analysis of tubulin solution. **(A-C)** Venn diagrams showing cross-links of 10 mM BS^3 **(A),** 0.1 wt% FA **(B)**, and 1 wt% FA **(C)**. **(D-F)** Connectivity maps of the common crosslinks. **(G-I)** Histograms of the distances that the common crosslinks span on the atomic structure of the tubulin (PDB ID 1JFF, [Löwe, 2001]). **(J-L)** Mapping of the crosslinks on the atomic structure. Alpha and beta tubulin subunits are colored blue and pink, respectively.

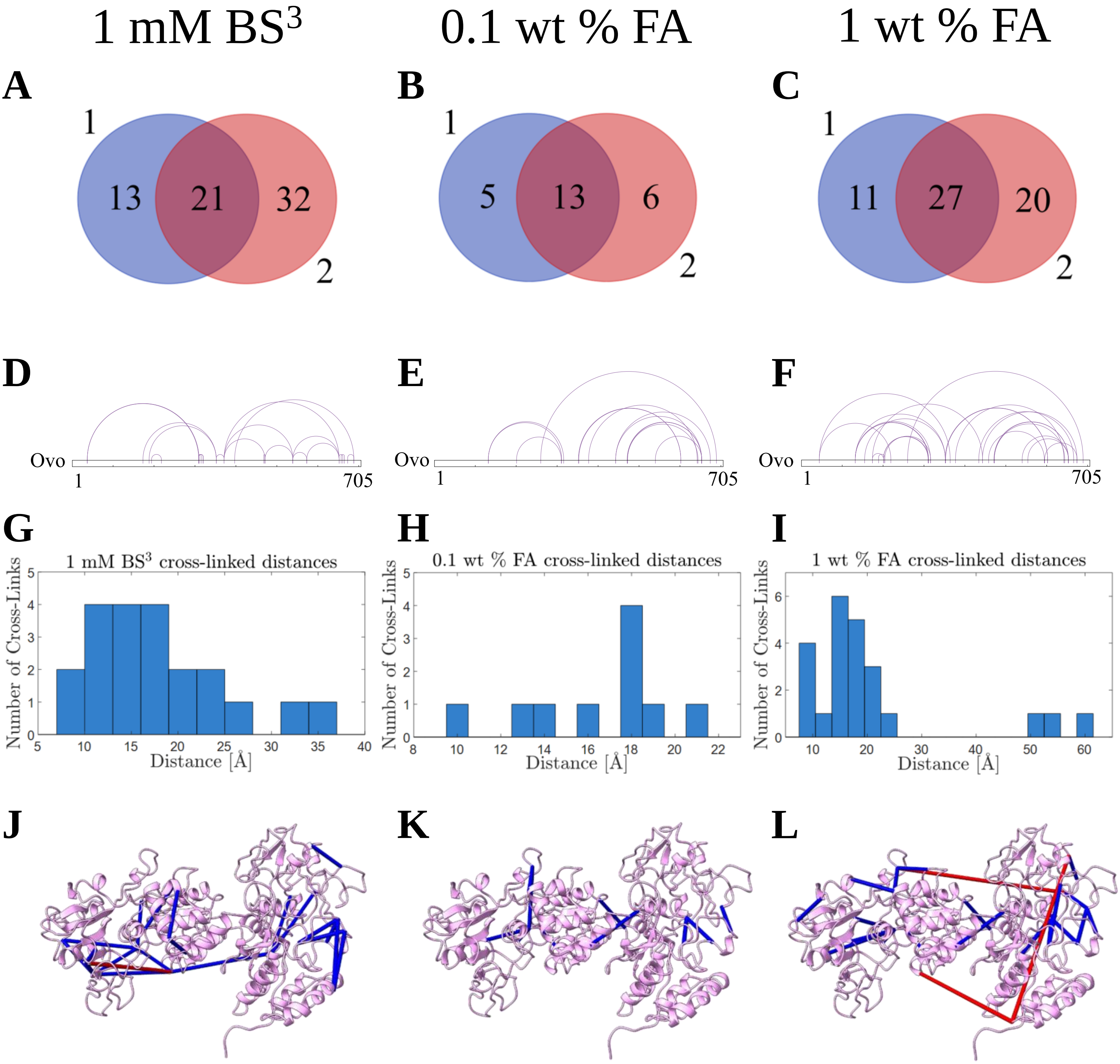

Figure S5. XL-MS analysis of Ovotransferin solution. **(A-C)** Venn diagrams showing cross-links of 1 mM BS^3 **(A),** 0.1 wt% FA **(B)**, and 1 wt% FA **(C)** in each experimental repeat. **(D-F)** Connectivity maps of the common crosslinks in the experimental repeats. **(G-I)** Histograms of the distances that the common crosslinks span on the atomic structure of Ovotransferin (PDB ID 1aiv [Kurokawa, 1999]). **(J-L)** Mapping of the crosslinks on the atomic structure. Crosslinks spanning a distance longer than 35 Å are in red.

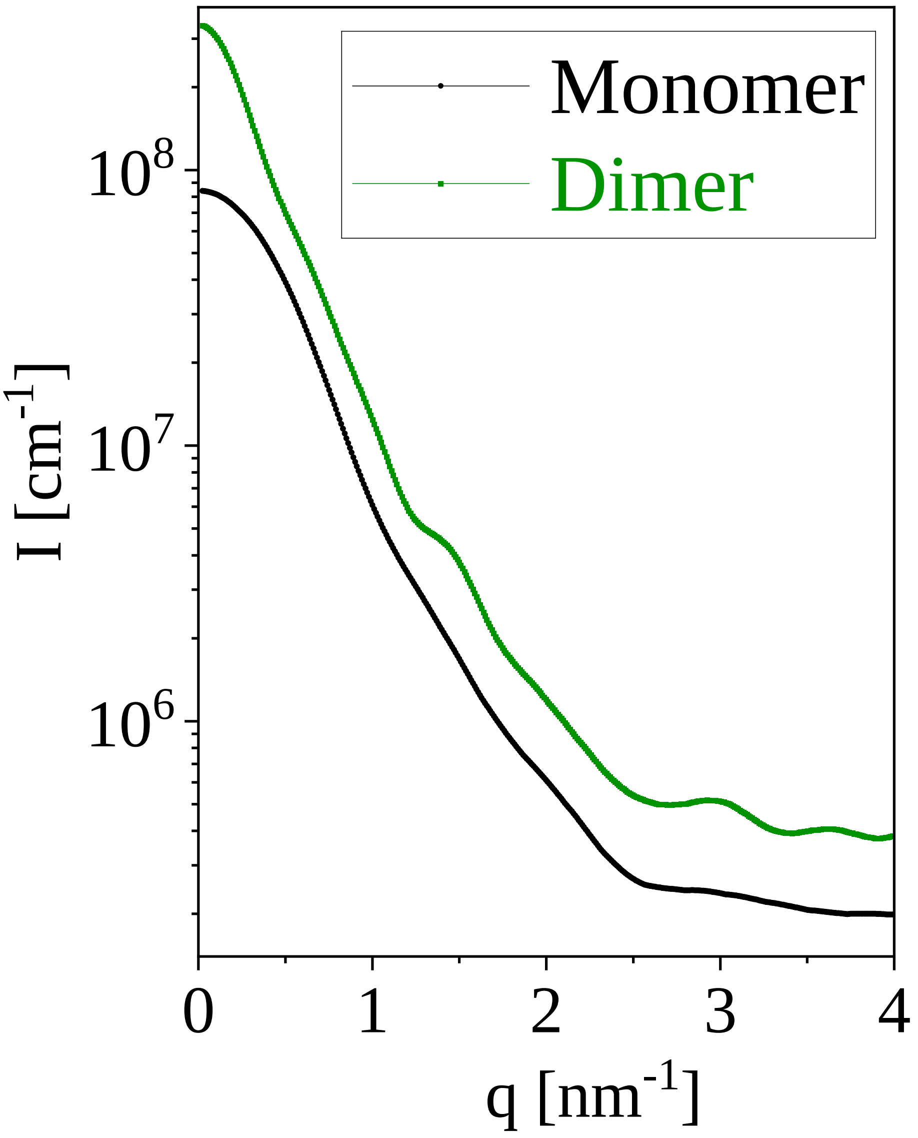

Figure S6. The computed scattering curves from models of Ovo. Using our in-house analysis software, D+, we computed the expected solution scattering curve from an atomic model of an Ovo monomer (PDB ID 1AIV [Kurokawa et al. 1999]) and a dimer. We created a dimer as follows: Each monomer measures approximately nine nanometers in length. By shifting the center of mass of the additional monomer by nine nanometers, we developed a dimer model that aligns with the SAXS spectra. For all the D+ computed models, the contribution of the monomer and dimer hydration layers were taken into account. The best fit to the data was obtained when the mass fractions of monomeric and dimeric OVO were 82 % and 18 %, respectively, as shown in Figure 6E.

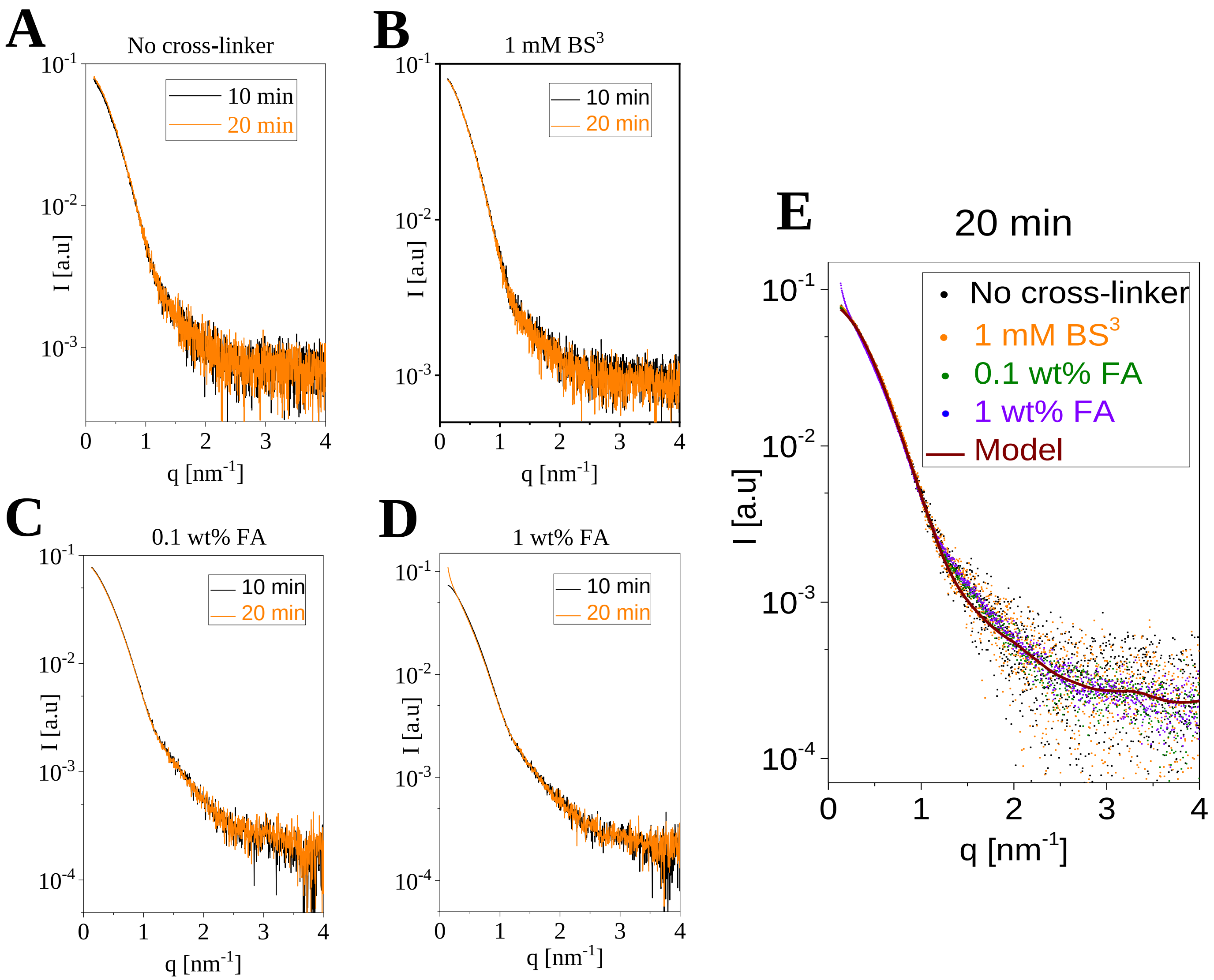

Figure S7. Effect of BS^3 and FA on the scattering curves from 8 mg/ mL bovine serum albumin (BSA) solution. BSA **(A)**, BSA crossed linked by 1 mM BS^3 **(B)**, 0.1 wt% FA **(C)**, or 1 wt% FA **(D),** following an incubation of 10 or 20 min. **(E)** Comparing the SAXS curve from BSA with the SAXS curves after 20 min incubation with 1 mM BS^3, 0.1, 1 wt% FA. The solid wine curve was computed by D+ software from the solvated atomic structure of BSA using PDB ID 4f5u [Bujacz, 2012] for the BSA monomer and 3v03 [Majorek, 2012] for the BSA dimer. D+ used a solvent voxel size of 0.2 nm, solvent probe radius of 0.14 nm, solvation thickness of 0.2 nm, and hydration layer electron density of 364 e^-/nm^3. The model that best fitted the data was a linear combination of 71 mol % monomers and 29 mol % dimers.

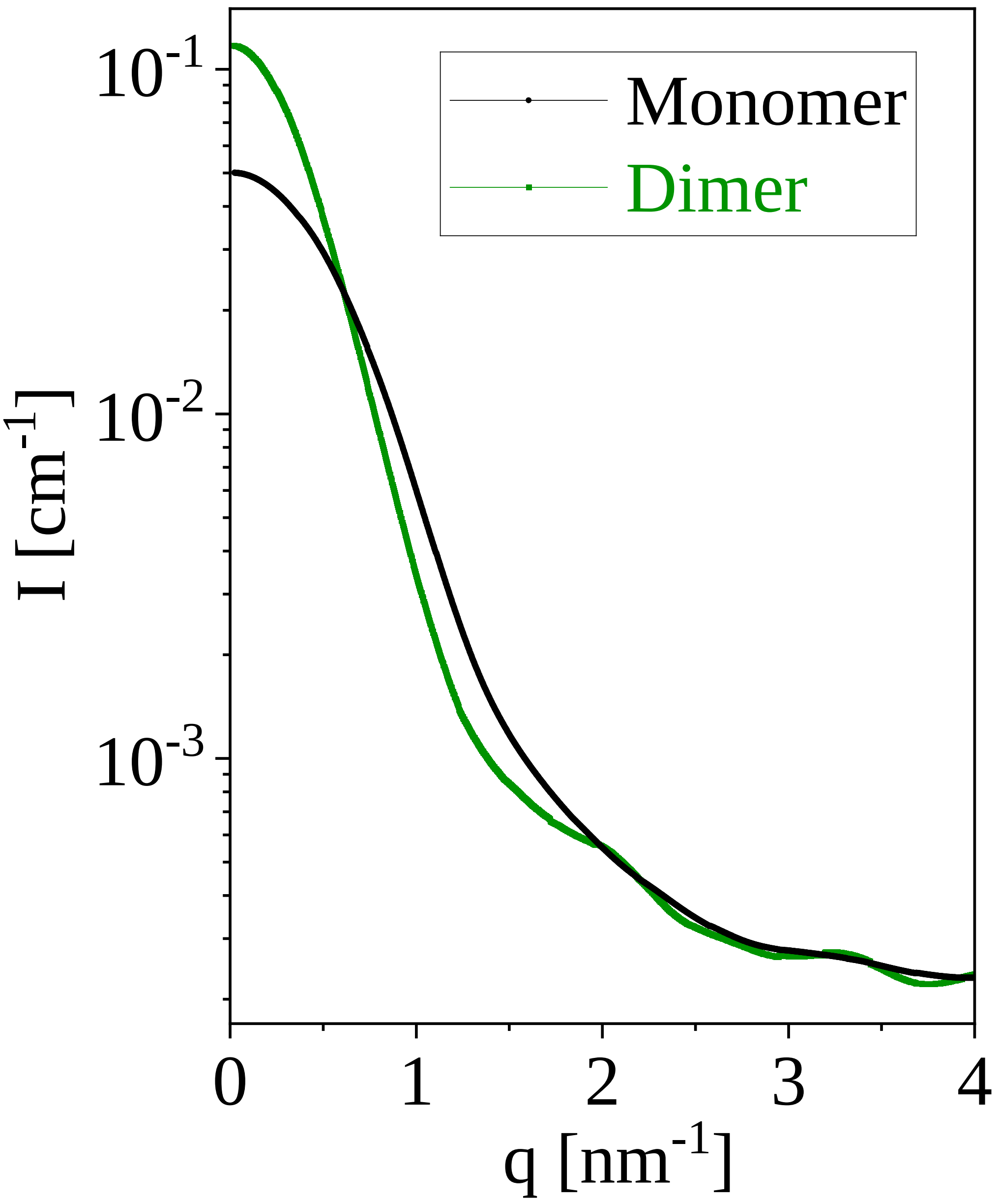

Figure S8. The computed scattering curves from models of BSA. Using our in-house analysis software, D+, we computed the expected solution scattering curve from an atomic model of a BSA monomer (PDB IDs 4f5u) and a dimer (PDB ID 3v03).

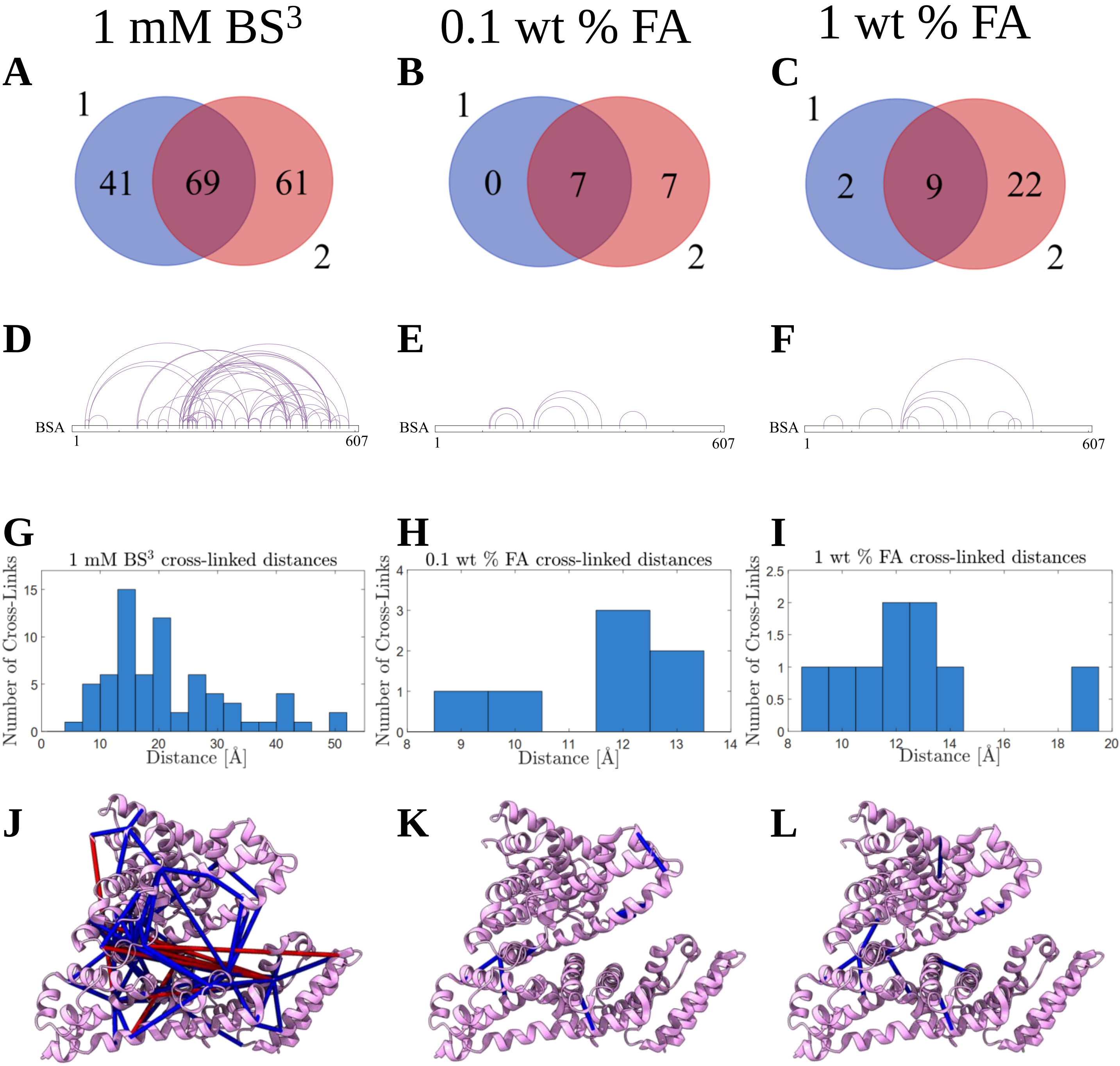

Figure S9. XL-MS analysis of BSA solution. **(A-C)** Venn diagrams showing cross-links of 1 mM BS^3 **(A),** 0.1 wt% FA **(B)**, and 1 wt% FA **(C)** in each experimental repeat. **(D-F)** Connectivity maps of the common crosslinks in the experimental repeats. **(G-I)** Histograms of the distances that the common crosslinks span on the atomic structure of BSA (PDB ID 4f5u [Bujacz, 2012]). **(J-L)** Mapping of the crosslinks on the atomic structure. Crosslinks spanning a distance longer than 35 Å are in red.
